# Spectrum and Management of Anorectal Disorders: A Retrospective Analysis From the United Arab Emirates

**DOI:** 10.7759/cureus.104155

**Published:** 2026-02-23

**Authors:** Chris M Prince, Adhya M Tom, Sofia Ali, Jagat S Gopinath, Ashish Enos, Anusha Sreejith

**Affiliations:** 1 School of Medicine, Georgian National University SEU, Tbilsi, GEO; 2 General Surgery, Gulf Medical University, Sharjah, ARE; 3 ENT, Gulf Medical University, Ajman, ARE; 4 Orthopedics, Gulf Medical University, Ajman, ARE; 5 General Surgery, Thumbay Hospital, Ajman, ARE; 6 Community Medicine, College of Medicine, Gulf Medical University, Ajman, ARE

**Keywords:** anal fissure, anal fistula, anorectal disorders, conservative management, hemorrhoids, surgical management

## Abstract

Background: Anorectal disorders, including hemorrhoids, anal fissures, and anal fistulas, are common conditions that can significantly impact quality of life. Data on their prevalence, clinical presentation, and management in the United Arab Emirates (UAE) are limited. This study aimed to evaluate the distribution, symptoms, and treatment patterns of anorectal disorders in a tertiary care setting in the UAE.

Methods: A retrospective review of medical records was conducted for 176 patients diagnosed with anorectal disorders at a tertiary hospital in the UAE, between January 2024 and January 2025. Sociodemographic data, clinical presentation, and management modalities were extracted using a structured proforma. Descriptive statistics were performed using SPSS version 29 (IBM Corp., Armonk, NY).

Results: A total of 176 patients were included, with a predominance of males (153, 86.9%) and individuals aged ≥35 years (97, 55.1%). Patients of Asian nationality accounted for 137 (77.8%), and 122 (69.4%) were overweight or obese. Hemorrhoids were the most common disorder, with 71 (40.8%) patients presenting with grade III and 26 (14.8%) with grade IV disease, followed by anal fissures in 49 (27.3%) patients and anal fistulas in 11 (6.3%) patients. The most frequent presenting complaint was a combination of pain and bleeding seen in 94 patients (53.4%), followed by bleeding alone in 62 patients (35.2%) and pain alone in 20 patients (11.4%). Surgical intervention was more common than conservative management, with hemorrhoidectomy performed in 75 (42.6%) cases, conservative treatment in 69 (39.2%), fissurectomy in 25 (14.2%), and fistulectomy in 7 (4.0%).

Conclusions: Hemorrhoids and anal fissures are the most prevalent anorectal disorders in this UAE tertiary care population, frequently presenting with pain and bleeding. Surgical intervention predominates, likely due to delayed presentation and advanced disease. Early detection, conservative management, and targeted public health strategies could reduce progression to severe disease and minimize the need for surgery. Training primary care providers to recognize anorectal disorders promptly may further improve patient outcomes.

## Introduction

Anorectal disorders include problems that are anatomical, neuromuscular, and functional. They are widespread, frequently upsetting, and occasionally incapacitating, and they greatly increase the cost of medical care. They often present with several overlapping symptoms, which can make it difficult to diagnose and treat the underlying illness [1]. A systematic review concluded that the prevalence of anorectal disorders in Africa and the Middle East could not be assessed, but it was highest in Australia and Oceania, followed by North America, Asia, and Europe. For 19 (24%), 46 (57%), and 15 (19%) studies, the risk of bias was low, moderate, and high, respectively [2].

The enlargement and distal displacement of the usual anal cushions are the symptoms of hemorrhoids, a relatively frequent anorectal ailment. They are a significant medical and economic issue that impacts millions of people worldwide [3]. Across an investigation conducted across Europe, eleven percent of the general population had hemorrhoidal disease, according to this global online survey study [4]. A study conducted in Ethiopia found that 13.1% (95% confidence interval (CI): 10.1-16.8) of the 403 study participants had hemorrhoids [5]. Analysis of the age distribution from a study in Kabul, which came from a retrospective research, showed that young people, usually between the ages of 30 and 40, were the age group most commonly afflicted with hemorrhoids [6].

According to a study from Saudi Arabia, overweight people had a 25.5% increased risk of hemorrhoids [7]. A national online survey revealed that common initial signs/symptoms were pain (60%), bleeding (47%), and discomfort (43%) [4]. A study from India reported that the most common clinical symptoms were bleeding per rectum and a mass per rectum, observed in 85% of patients, followed by pain during defecation in 77.5% [8]. A study showed that surgical treatments provide superior symptom relief, faster recovery, and lower recurrence but with some specific post-treatment complications, while conservative treatments are safer and less invasive, which provides slower symptom relief and higher recurrence rates [9].

A systematic review concluded that a total of 26 studies providing data estimated a total prevalence of anal fistulas to be 1.69 per 10,000 population [10]. Similarly, another review showed that the overall prevalence of anal fistula in European countries was 8.37 (95% CI: 18.20%-18.55%) per 100,000 individuals, and the highest prevalence was reported for Italy [11].

Results from an investigation from India concluded that 44% of patients with anal fistula were in the age group of 31-40 years [12]. From China, the findings showed that out of the 1,783 cases with anal fistula, 1,526 were males with a median age of 36 years, while 257 were females with a median age of 35 years, yielding a male-to-female ratio of 5.9:1 [13].

Topical medications, including local anesthetics, vitamins, or anti-inflammatory agents, are commonly used, though none have proven superior to simple lubricants, and anesthetic-containing preparations show no benefit over placebo [14]. Suppositories can aid stool passage, while analgesics such as nonsteroidal anti-inflammatory drugs (NSAIDs), acetaminophen, or opioids help relieve pain. Conservative treatment for three weeks heals nearly 50% of acute anal fissures, but recurrence is common if underlying causes persist or stool softeners are stopped prematurely, necessitating prolonged therapy [15].

A study conducted in the United States reported 1,243 cases of anal fissures, comprising 721 females (58%) and 522 males (42%). The prevalence varied significantly by age group, ranging from 0.05% in patients aged 6-17 years to 0.18% in those aged 25-34 years [16]. In an Indian study, 629 patients were enrolled with a mean age of 38.27 ± 9.25 years. The majority were male (438, 69.63%), and nearly half (308, 48.97%) had a normal body mass index (BMI). Anal fissures were diagnosed in 112 patients, accounting for 17.81% of the study population [17]. A cross-sectional study conducted in Mali concluded that symptoms were predominantly proctalgia (84%), followed by rectal pain (24%), constipation (12%), anal discharge (8%), and anal pruritus (0.71%) [18].

In a review of 876 patients with anal fissures, pain was identified as the most frequent symptom, affecting 90.8% of cases. Patients typically report sharp or tearing pain associated with defecation, which may occur only during bowel movements or persist for several minutes to hours afterward. Bleeding is another common symptom, observed in 71.4% of patients [19].

Another search revealed that a total of 1,532 patients were analyzed. Pooled data showed that 1,117 patients (72.7%) achieved fissure healing following the first botulinum toxin injection (*P* < 0.001, *I*² = 86.6%) [20]. A retrospective study concluded that in 100 patients, 29 patients underwent fissurectomy and 91 lateral internal sphincterotomy [21].

This study aims to evaluate anorectal disorders at a tertiary care center in the United Arab Emirates (UAE), focusing on their proportion, the most commonly diagnosed conditions, and patterns of medical management. The findings will help fill regional knowledge gaps, guide evidence-based clinical practice, and support the development of standardized management strategies tailored to the UAE population.

## Materials and methods

Study area and period

This study was conducted at a Tertiary Hospital in the UAE. Medical records of 176 patients diagnosed with anorectal disorders between January 2024 and January 2025 were reviewed.

Study design and study population

An institution-based retrospective record review study design was employed. The study population comprised all patients diagnosed with anorectal disorders during the study period at the Tertiary Hospital in the UAE.

Eligibility criteria

Patients with a confirmed diagnosis of anorectal disorders recorded between January 2024 and January 2025 were included in the study. Medical records with incomplete or missing data were excluded from the analysis.

Sample size and study variables

The sample included all patients diagnosed with anal fissure, anal fistula, and hemorrhoids at Tertiary Hospital, Ajman, from January 2024 to January 2025 who fulfilled the inclusion criteria. The study variables included sociodemographic characteristics, presenting symptoms, laboratory parameters, and management modalities related to anorectal disorders.

Data collection tool and procedure 

A structured proforma was developed to extract relevant information from the hospital’s electronic medical records. The proforma captured sociodemographic details, clinical presentation, and management-related variables. Three physicians conducted content validation of the proforma to ensure adequacy and relevance. Following ethical approval from the Institutional Review Board (IRB) of Gulf Medical University, administrative permission was obtained from the hospital’s medical director before data extraction. A pilot study was conducted using records of five patients to assess the feasibility and clarity of the data collection tool.

Data management and analysis

Collected data were entered into Microsoft Excel and subsequently analyzed using SPSS version 29 (IBM Corp., Armonk, NY). Descriptive statistics, including frequencies, percentages, means, and standard deviations, were used where appropriate, along with chi-square analysis for association.

Ethical clearance

Ethical approval was obtained from the Institutional Review Board of Gulf Medical University. Administrative consent to conduct the study was secured from the Hospital Director. Patient confidentiality was maintained by anonymizing all identifying information. Access to the data was restricted to the investigators, IRB members, and the statistician. As the study was solely record-based, it posed no direct risk to participants.

## Results

A total of 176 patients diagnosed with anorectal disorders were included in the study. The analysis focused on sociodemographic characteristics, types of anorectal disorders, presenting symptoms, and treatment modalities. Descriptive statistics were used to summarize the distribution of variables across the study population.

As shown in Table 1, more than half of the patients (97, 55.1%) were aged 35 years or older, while 79 (44.9%) were below 35 years of age. The study population was predominantly male (153, 86.9%), with females accounting for only 23 (13.1%) of cases. The majority of patients were of Asian nationality (137, 77.8%), followed by African (19, 10.8%) and Middle Eastern (17, 9.7%) origins. Regarding BMI, 67 (38.1%) patients were overweight, 55 (31.3%) were obese, and 54 (30.7%) had a normal BMI.

**Table 1:** Sociodemographic factors of patients diagnosed with anorectal disorders (n = 176). BMI, body mass index

Variables		Frequency (*n*)	Percentage (%)
Age	Less than 35	79	44.9
	More than or equal to 35	97	55.1
Gender	Female	23	13.1
	Male	153	86.9
Nationality	Middle East	17	9.7
	Asia	137	77.8
	Africa	19	10.8
	Europe and North America	3	1.7
BMI	Normal weight (<24.9 kg/m²)	54	30.7
	Overweight (25.0-29.9 kg/m²)	67	38.1
	Obese (>30.0 kg/m²)	55	31.3

**Figure 1:**
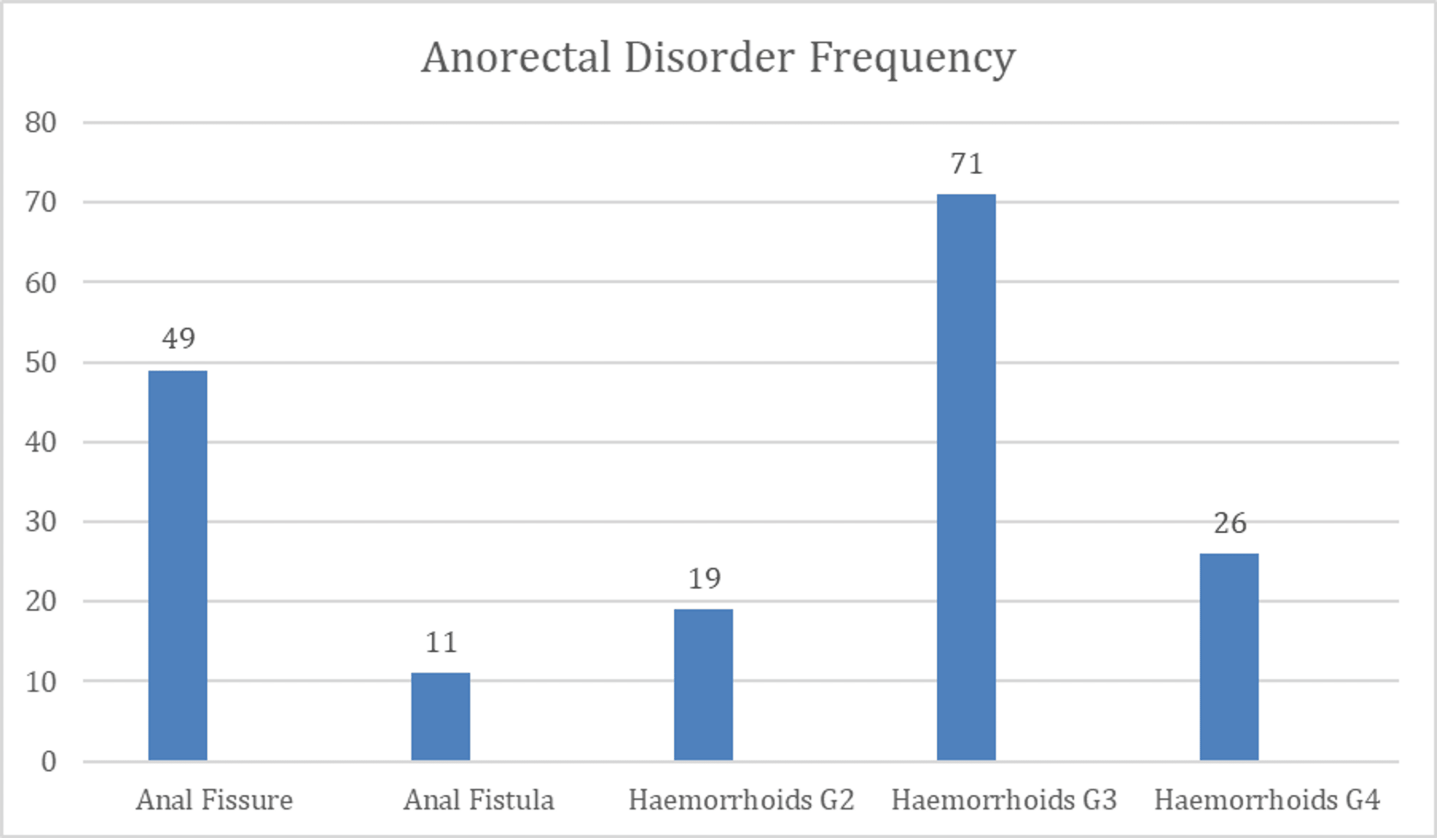
Types of anorectal disorders seen in patients (n = 176).

Figure 1 illustrates the distribution of anorectal disorders among the patients. Hemorrhoids were the most common condition, with grade III hemorrhoids accounting for the largest proportion (40.8%), followed by grade IV hemorrhoids (14.8%) and grade II hemorrhoids (10.8%). Anal fissures were identified in 49 (27.3%) patients, while anal fistulas were less frequent, affecting 11 (6.3%) of the study population.

According to Table 2, the most common presenting complaint was a combination of pain and bleeding, reported by 94 (53.4%) patients. Bleeding alone was noted in 62 (35.2%) cases, while pain alone was reported by 20 (11.4%). In terms of management, surgical intervention was more common than conservative treatment. Hemorrhoidectomy was the most frequently performed procedure (75, 42.6%), followed by conservative management (69, 39.2%), fissurectomy (25, 14.2%), and fistulectomy (7, 4%).

**Table 2:** Presenting symptoms and treatment of anorectal disorders (n = 176).

Variables		Frequency (*n*)	Percentage (%)
Presenting symptoms	Pain	20	11.4
	Bleeding	62	35.2
	Pain and bleeding	94	53.4
Treatment	Conservative	69	39.2
	Fissurectomy	25	14.2
	Fistulectomy	7	4
	Hemorrhoidectomy	75	42.6

As given in Table 3, a statistically significant association was observed between age and the type of anorectal disorder (*P* = 0.007). Among patients aged ≤35 years, anal fissure was more common (31, 39.2%), followed by hemorrhoids (45, 57%), while anal fistula was relatively uncommon (3, 3.8%). In contrast, patients aged >35 years showed a higher prevalence of hemorrhoids (71, 73.2%) and anal fistula (8, 8.2%), with fewer cases of anal fissure (18, 18.6%). These findings suggest that younger individuals are more likely to present with anal fissures, whereas hemorrhoids and anal fistulas are more prevalent in older age groups.

**Table 3:** Association between age and type of anorectal disorders. *P*-values from chi-square analysis.

Sociodemographic factors		Anorectal disorders			*P*-value
		Anal fissures, *n* (%)	Anal fistulas, *n* (%)	Hemorrhoids, *n *(%)	
Age	Less than or equal to 35 years	31 (39.2)	3 (3.8)	45 (57)	0.007
	More than 35 years	18 (18.6)	8 (8.2)	71 (73.2)	

## Discussion

Anorectal disorders represent a common yet often underreported clinical problem, with a significant impact on patient quality of life. Understanding the demographic characteristics, clinical presentation, and management approaches associated with these conditions is essential for improving diagnostic accuracy and optimizing treatment outcomes. This discussion contextualizes the study findings within existing literature and explores their clinical and public health implications.

In the present UAE-based study, more than half of the patients were aged ≥35 years (97, 55.1%), with a marked male predominance (153, 86.9%). Most patients were of Asian nationality (137, 77.8%), and a high proportion were overweight or obese (122, 69.4%). These findings are partly consistent with reports from other countries. An Indian cross-sectional study of 629 patients reported a similar mean age (~38 years) and male predominance (69.6%), although a larger proportion of patients had normal BMI, with constipation and physical inactivity identified as key risk factors [17]. Likewise, a Turkish study involving 211 patients demonstrated a higher mean age (~46 years) and male predominance (76.3%), but found no significant difference in BMI compared to controls [22]. In contrast, a large population-based study reported a slightly higher prevalence of hemorrhoids among females, with older age and increased BMI being significant risk factors, and associations varying by sex and parity [23]. Overall, the variations observed across studies may be attributed to differences in population demographics, lifestyle factors, and healthcare-seeking behaviors. The high prevalence of overweight and obesity and the predominance of expatriate males in the UAE suggest a need for targeted public health interventions focusing on lifestyle modification, dietary counseling, and early identification of anorectal disorders.

In this study, hemorrhoids were the most prevalent anorectal disorder, with grade III hemorrhoids constituting the largest proportion (71, 40.8%), followed by grade IV (26, 14.8%) and grade II hemorrhoids (19, 10.8%). Anal fissures were identified in 49 (27.3%) patients, while anal fistulas were relatively uncommon, affecting 11 (6.3%) of the study population. These findings align with existing literature identifying hemorrhoidal disease as the predominant benign anorectal condition in clinical practice. A large review and a proctology clinic-based study similarly reported hemorrhoids as the most frequent anorectal pathology among symptomatic patients [24]. The predominance of advanced hemorrhoid grades in our cohort contrasts with community-based studies reporting mainly early-stage disease [5], suggesting delayed healthcare-seeking behavior, underrecognition of early symptoms, or referral bias toward more severe cases in tertiary care settings. This highlights the need for improved patient education, early screening, and timely referral from primary care to prevent disease progression.

The substantial proportion of patients with anal fissures in our study further supports evidence that fissures commonly coexist with hemorrhoidal disease, as previously reported in large clinical series [25]. In contrast, population-based data demonstrate that anal fistulas are rare in the general population [26], emphasizing that their presence in hospital-based cohorts likely reflects more complex or advanced anorectal pathology requiring specialist care. Regional variations observed in studies from Sudan [27] and other settings [28] underscore the influence of demographic, dietary, and healthcare-access factors on disease distribution.

From a clinical perspective, these findings suggest that proactive strategies focusing on early symptom recognition, dietary modification, bowel habit optimization, and conservative management at initial presentation may reduce progression to advanced hemorrhoidal disease and associated complications. Strengthening primary care training in the early diagnosis of anorectal disorders and increasing public awareness regarding anorectal symptoms could improve outcomes and reduce the burden of advanced disease requiring surgical intervention.

In the present study, the most common presenting complaint among patients with anorectal disorders was a combination of pain and bleeding, reported by 94 (53.4%) patients. Bleeding alone was noted in 62 (35.2%) cases, while pain alone was reported by 20 (11.4%). These findings are consistent with a clinical study from Rajasthan, which identified bleeding per rectum as the most frequent presenting symptom (55.45%), followed by pain during defecation (43.63%) [29]. Similarly, another observational review reported bleeding and anal pain as the predominant symptoms in patients with anorectal diseases, emphasizing that combined symptomatology is frequently encountered in clinical practice [30]. Clinically, the predominance of pain and bleeding highlights the importance of prompt evaluation to ensure early diagnosis and management. Enhancing public awareness and strengthening primary care training in early anorectal assessment may reduce delayed presentation and disease progression.

In this study, surgical intervention was more common than conservative management, with hemorrhoidectomy being the most frequently performed procedure (75, 42.6%), followed by conservative treatment (69, 39.2%), fissurectomy (25, 14.2%), and fistulectomy (7, 4%). These findings align with other clinical series reporting a predominance of surgical management for symptomatic anorectal conditions in tertiary practice, where definitive procedures such as hemorrhoidectomy and fissure/fistula surgery are often required in refractory or advanced cases [31,4]. Meta-analytic evidence also suggests that surgical treatment can offer superior short-term relief and lower recurrence for hemorrhoidal disease compared with conservative therapies, potentially contributing to its frequent use [9].

However, contradicting evidence highlights that conservative strategies such as dietary counseling, fiber supplementation, and topical medical therapy provide significant symptomatic improvement and remain the recommended first-line approach for early or less severe disease, with surgery reserved for failure of medical management or advanced pathology [32]. The high rate of surgical intervention in this study likely reflects the predominance of advanced or refractory cases, suggesting that many patients present late in the disease course. Early detection and timely conservative management, including dietary modifications, fiber supplementation, and lifestyle counseling, could reduce the need for surgery. Additionally, training primary care providers to recognize anorectal disorders early may improve outcomes and limit progression to severe disease requiring operative treatment.

This study has several limitations that should be acknowledged. First, its retrospective design and reliance on medical record data may have introduced information bias due to incomplete documentation or variability in clinical recording. Additionally, as the study was conducted in a single tertiary care center, the findings may not be fully generalizable to the broader UAE population. The predominance of expatriate males and the referral-based nature of the institution may have resulted in selection and referral bias, potentially overrepresenting advanced or symptomatic cases while underestimating milder conditions managed in primary care settings.

## Conclusions

Anorectal disorders represent a significant yet often underrecognized health burden in the UAE, with hemorrhoids and anal fissures being the most prevalent conditions. Patients predominantly present with pain and bleeding, which underscores the importance of early recognition and timely management. The high rate of surgical intervention, particularly hemorrhoidectomy, reflects the advanced stage of disease at presentation, highlighting gaps in early detection and conservative care. Strengthening primary care capacity for early diagnosis, promoting public awareness of anorectal symptoms, and implementing lifestyle and dietary interventions could reduce progression to severe disease and the need for invasive procedures. These findings provide valuable insight into the regional epidemiology and management patterns of anorectal disorders and can inform evidence-based strategies to improve patient outcomes and optimize healthcare resource utilization.
